# Precision oncology of lung cancer: genetic and genomic differences in Chinese population

**DOI:** 10.1038/s41698-019-0086-1

**Published:** 2019-05-03

**Authors:** Hongbing Shen, Meng Zhu, Cheng Wang

**Affiliations:** 1 Department of Epidemiology, School of Public Health, Nanjing Medical University, Nanjing, China; 2 Jiangsu Key Lab of Cancer Biomarkers, Prevention and Treatment, Collaborative Innovation Center for Cancer Medicine, Nanjing Medical University, Nanjing, China

**Keywords:** Lung cancer, Oncogenesis

## Abstract

Knowledge of the lung cancer genome has experienced rapid growth over the past decade. Genome-wide association studies and sequencing studies have identified dozens of genetic variants and somatic mutations implicated in the development of lung cancer in both Chinese and Caucasian populations. With the accumulating evidence, heterogeneities in lung cancer susceptibility were observed in different ethnicities. In this review, the progress on germline-based genetic variants and somatic-based genomic mutations associated with lung cancer and the differences between Chinese and Caucasian populations were systematically summarized. In the analysis of the genetic predisposition to lung cancer, 6 susceptibility loci were shared by Chinese and Caucasian populations (3q28, 5p15, 6p21, 9p21.3, 12q13.13 and 15q25), 14 loci were specific to the Chinese population (1p36.32, 5q31.1, 5q32, 6p21.1, 6q22.2, 6p21.32, 7p15.3, 10p14, 10q25.2, 12q23.1, 13q22, 17q24.3, 20q13.2, and 22q12), and 12 loci were specific to the Caucasian population (1p31.1, 2q32.1, 6q27, 8p21.1, 8p12, 10q24.3, 11q23.3, 12p13.33, 13q13.1, 15q21.1, 20q13.33 and 22q12.1). In the analysis of genomic and somatic alterations, different mutation rates were observed for *EGFR* (Chinese: 39–59% *vs*. TCGA: 14%), *KRAS* (Chinese: 7–11% *vs*. TCGA: 31%), *TP53* (Chinese: 44% *vs*. TCGA: 53%), *CDKN2A* (Chinese: 22% *vs*. TCGA: 15%), *NFE2L2* (Chinese: 28% *vs*. TCGA: 17%)*, STK11* (Chinese: 4–7% *vs*. TCGA: 16%), *KEAP1* (Chinese: 3–5% *vs*. TCGA: 18%), and *NF1* (Chinese: <2% *vs*. TCGA: 12%). In addition, frequently amplified regions encompassing genes involved in cytoskeletal organization or focal adhesion were identified only in Chinese patients. These results provide a comprehensive description of the genetic and genomic differences in lung cancer susceptibility between Chinese and Caucasian populations and may contribute to the development of precision medicine for lung cancer treatment and prevention.

## Introduction

Lung cancer has been the most frequently diagnosed cancer and the leading cause of cancer death worldwide for several decades.^1^ In China, it was estimated that there were 0.73 million new cases and 0.61 million deaths from lung cancer in 2015, accounting for more than one-third of the world total.^2^ Although tobacco smoking has been recognized as the most important risk factor associated with lung cancer, familial aggregations have been observed in lung cancer patients even among nonsmokers, suggesting an important role of heredity in the development of lung cancer.^3,4^ The heritability of lung cancer was recently estimated to be 15.2% in a Chinese population.^5^

Although heredity has long been implicated in the development of lung cancer, the germline-based genetic variants and somatic-based genomic mutations associated with lung cancer have just begun to be revealed in recent decades.^6^ With the accumulation of these findings, genetic and genomic heterogeneity associated with different histopathological types of lung cancer, as well as among different ethnic groups, was observed. The most representative finding is the L858R mutation in the EGFR, which was more frequently detected in Asian patients with adenocarcinoma histology.^7^ Such heterogeneities are widespread in lung cancer, but a systematic summary of the results has been lacking until now. Here, the main findings of genetic and genomic studies of lung cancer and the differences associated with lung cancer in Chinese and Caucasian patients were reviewed.

The success of the International HapMap Project provided a foundation for genomic epidemiological studies.^8^ After its completion, researchers began to identify susceptibility genes and genetic variations associated with lung cancer based on association studies. Variants in candidate susceptibility genes, such as genes involved in DNA damage and repair pathways, the cell cycle pathway, and the inflammatory pathway, as well as genes involved in the detoxification or metabolic activation of carcinogens and tobacco smoke, have been widely studied in Chinese populations.^9–12^ However, many of these associations are controversial and show low verification rates in different studies.

### Findings from lung cancer GWAS in Chinese populations

With the development and application of high-throughput genotyping technologies, genome-wide association studies (GWAS) have proven to be an efficient way to explore the genetic predisposition of complex traits.^13^ In 2011, Hu, Z. et al. conducted the first GWAS of lung cancer in a Chinese population, a large-scale multistage case-control study.^14^ A total of 906,703 single-nucleotide polymorphisms were genotyped in 2,383 lung cancer cases and 3,160 controls followed by a fast-track validation using 6,313 cases and 6,409 controls^14^ and a second round validation in an enlarged sample of 7,436 cases and 7,483 controls.^15^

Together, these two studies identified seven loci at the genome-wide significance level (*P* < 5.0 × 10^−8^); they included rs4488809 at 3q28 (*TP63*), rs2736100 and rs465498 at 5p15 (*TERT-CLPTM1L*), rs2895680 at 5q32 (*STK32A-DPYSL3*), rs1663689 at 10p14 (*GATA3*), rs753955 at 13q22 (*MIPEP-TNFRSF19*), rs4809957 at 20q13.2 (*CYP24A1*), and rs17728461 and rs36600 at 22q12 (*MTMR3-HORMAD2*). In addition, rs9439519 at 1p36.32 (*AJAP1-NPHP4*) and rs247008 at 5q31.1 (*IL3-CSF2*) were consistently associated with the risk of lung cancer at different stages. Of the identified variants, rs2736100, rs2895680, rs4809957, rs247008, and rs9439519 showed evidence of interaction with smoking dose.

Adenocarcinoma (AC) and squamous cell carcinoma (SqCC) are two major histological subtypes of lung cancer.^16^ Obvious heterogeneity has been observed among different histological subtypes of lung cancer; for example, 3q28 (*TP63*) and 5p15 (TERT-CLPTM1L) were more prominent among AC,^17,18^ and 12q23.1 (*SLC17A8*-*NR1H4*) was significantly associated only with SqCC. These findings represent a considerable advance in the understanding of lung cancer susceptibility in the Chinese population.^19^

### Exploring the missing heritability of lung cancer in the Chinese population

Although lung cancer GWAS in Chinese populations have identified several robust susceptibility loci, only a fraction of the heritability of lung cancer susceptibility can be explained by these variants.^5^ To explore the “missing heritability”, a series of explorations was performed that included studies of the pleiotropy of susceptibility genes in multiple types of cancer,^20^ genome-wide meta-analysis in never-smoking women,^21,22^ and the association of low-frequency variants with lung cancer occurrence.^23^

Evidence for the pleiotropy of genes/loci had been obtained in previous GWAS^24,25^ and is biologically plausible. Jin G et al. mapped genetic variants that have consistent effects on risk of multiple cancers using genome-wide scan data on lung cancer, noncardia gastric cancer, and esophageal squamous cell carcinoma obtained from 5,368 cases and 4,006 controls followed by an evaluation in an additional 9,001 cases and 11,436 controls.^20^ Consistent associations of rs2494938 at 6p21.1 (*LRFN2*) and rs2285947 at 7p15.3 (*SP4*) were observed with these three cancers. The minor alleles of rs2494938 and rs2285947 were significantly associated with an increased risk of lung cancer in this Chinese population.

In China and throughout Asia, most women are nonsmokers.^26^ However, an obvious increase of lung cancer incidence (especially that of AC) was observed in never-smoker women recently. In a GWAS conducted by the Female Lung Cancer Consortium in Asia (FLCCA), 66,09 never-smoking female lung cancer cases and 7,457 controls drawn from 14 studies were analyzed (4,839 cases and 5,050 controls were from China).^21^ In addition to confirming the associations reported for loci at 3q28, 5p15.33, and 17q24.3, the study also identified three new susceptibility loci at 6p21.32 (rs2395185, *HLA Class II region*), 6q22.2 (rs9387478, *ROS1*-*DCBLD1*), and 10q25.2 (rs7086803, *VTI1A*). A subsequent meta-analysis with an extended sample size of the FLCCA reported another three novel loci, including 6p21.1 (rs7741164, *DQ14194*), 9p21.3 (rs72658409, *CDKN2B*), and 12q13.13 (rs11610143, *ACVR1B*).^22^

GWAS usually focus on common genetic variants (variants with minor allele frequency (MAF) ≥ 0.05) while neglecting low-frequency or rare variants (MAF < 0.05).^27^ The Illumina Human Exome Beadchip, which mainly focuses on low-frequency and rare missense variants in exon regions, was recently developed.^28^ Using this chip, Jin, G. et al. scanned 1,348 lung cancer cases and 1,998 control subjects and subsequently evaluated promising associations in an additional 4,699 affected subjects and 4,915 control subjects.^23^ Three low-frequency missense variants, *BAT2* (rs9469031, c.1544C>T [p.Pro515Leu]), *FKBPL* (rs200847762, c.410C>T [p.Pro137Leu]), and *BPIFB1* (rs6141383, c.850G>A [p.Val284Met]), were observed associated with lung cancer risk. Rs9469031 in *BAT2* and rs6141383 in *BPIFB1* were also associated with the age of onset of lung cancer (*P* = 0.001 and 0.006, respectively). These findings extend the genetic associations of GWAS and help explain the additional heritability of lung cancer in the Chinese population.

### Similarities and differences in lung cancer susceptibility between Chinese and Caucasian populations

In 2008, three independent studies simultaneously reported chromosome 15q25.1 as a susceptibility region for lung cancer in Caucasian populations; in this region, rs8034191 and rs1051730 were the major tagging variants.^29–31^ The region harbors six protein-coding genes, including three genes, *CHRNA3*, *CHRNB4*, and *CHRNA5*, that encode nicotinic acetylcholine receptors. A subsequent study found that rs1051730 near *CHRNA3* was strongly associated with smoking quantity and nicotine addiction as well as with lung cancer risk.^30^ With an enlarged sample size and pooling analysis, rs402710 and rs2736100 at 5p15.33 (*CLPTM1L*-*TERT*) were shown to be independently associated with lung cancer risk (*r*^2^ = 0.026).^32–34^ *TERT* encodes telomerase reverse transcriptase, which plays an important role in maintaining the length and stability of human telomeres. *CLPTM1L* was overexpressed in non-small cell lung cancer and protected tumor cells from genotoxic stress-induced apoptosis.^35^ RNA interference-mediated blockade of *CLPTM1L* could inhibit K-Ras-induced lung tumorigenesis.^36^ Using a higher-density chip, Wang, et al. validated the associations at 5p15.33 and 15q25.1 and identified a novel locus at 6p21.33 (rs3117582, *BAT3*-*MSH5*).^37^ In 2010, a meta-analysis combining 16 GWAS confirmed the susceptibility loci at 15q25.1, 5p15.33, and 6p21.33. Stratification analysis identified three novel loci for SqCC, including 2q32.1 (rs11683501, *NUP35*), 9p21.3 (rs1333040, *CDKN2A*- *CDKN2B*), and 12p13.33 (rs10849605, *RAD52*).^38^ In 2014, another GWAS meta-analysis tested the associations of less frequent variants using imputation based on the 1000 Genomes Project. In this study, two rare variants of *BRCA2* (rs11571833, p.Lys3326X) and *CHEK2* (rs17879961, p.Ile157Thr) were identified with large-effect genome-wide associations for SqCC.^39^ In 2017, the OncoArray Consortium genotyped 14,803 additional lung cancer cases and 12,262 additional controls using the OncoArray in Caucasian populations.^40^ After integration with the existing GWAS data, 29,266 cases and 56,450 controls were used in an association analysis. This study highlighted the genetic heterogeneity across histological subtypes of lung cancer and reported novel loci for lung cancer at 1p31.1 (rs71658797, *FUBP1*), 6q27 (rs6920364, *RNASET2*), 8p21.1 (rs11780471, *CHRNA2*), and 15q21.1 (rs66759488, *SEMA6D*). Stratification by histology identified five novel loci for AC, including 8p12 (rs4236709, *NRG1*), 10q24.3 (rs11591710, *OBFC1*), 11q23.3 (rs1056562, *AMICA1*), 15q21.1 (rs77468143, *SECISBP2L*) and 20q13.33 (rs41309931, *RTEL1*).

Comparing the findings of GWAS for different ethnic groups, genetic susceptibility to lung cancer showed both similarities and differences in Chinese and Caucasian populations (Table 1).^41^ The identified regions 3q28, 5p15, 6p21, and 9p21.3 are shared by the two ethnic populations. However, although 6p21 was a shared susceptibility region, rs3117582 was nonpolymorphic in the Chinese population, whereas the low-frequency missense variants rs9469031 and rs200847762 were associated with lung cancer risk in Chinese.^23^ In a two-stage study of never smokers exposed to second-hand smoke, an intronic SNP, rs12809597, in the *ACVR1B* gene (12q13.13) was also found to be associated with the risk of lung cancer in Caucasians, suggesting that 12q13.13 is a shared susceptibility locus.^42^ In the fine-mapping analysis of 15q25, which was associated with smoking quantity and lung cancer in Caucasians, the strongest susceptibility variants rs8034191 and rs1051730 were rare (MAF < 0.05) in the Chinese population.^43^ Further analysis identified four common variants (rs2036534, rs667282, rs12910984 and rs6495309) at 15q25 that are associated with lung cancer risk in the Chinese population. In addition to the shared susceptibility loci described above, 14 loci (1p36.32, 5q31.1, 5q32, 6p21.1, 6q22.2, 6p21.32, 7p15.3, 10p14, 10q25.2, 12q23.1, 13q22, 17q24.3, 20q13.2, and 22q12) specific to the Chinese population and 12 loci (1p31.1, 2q32.1, 6q27, 8p21.1, 8p12, 10q24.3, 11q23.3, 12p13.33, 13q13.1, 15q21.1, 20q13.33 and 22q12.1) specific to the Caucasian population were reported^41^ (Table 1).

**Table 1:** Susceptibility loci for lung cancer identified by GWAS in Chinese and Caucasian populations

	Region	Gene	Disease/trait^a^	Key SNPs	Risk Allele	OR	*P*-values	Refference
Shared								
	3q28	*TP63*	NSCLC	rs4488809	C	1.26 (1.21–1.32)	7.20 × 10^−26^	Hu, Z.^14^
			AC	rs13080835	G	1.12 (1.08–1.15)	7.50 × 10^−12^	McKay, J.D.^40^
			AC	rs13314271	T	1.13 (1.09–1.18)	7.20 × 10^−10^	Wang, Y.^39^
	5p15	*TERT- CLPTM1L*	LC	rs401681	G	1.15 (1.09–1.19)	7.90 × 10^−9^	Wang, Y.^37^
			LC	rs4975616	G	1.15 (1.10–1.20)	3.00 × 10^−9^	Broderick, P.^33^
			NSCLC	rs2736100	C	1.27 (1.22–1.33)	1.00 × 10^−27^	Hu, Z.^14^
			AC	rs31489	C	1.24 (1.17–1.31)	3.70 × 10^−14^	Landi, M.T.^34^
			AC	rs7705526	A	1.25 (1.21–1.29)	3.80 × 10^−35^	McKay, J.D.^40^
	6p21	*BAT3*	LC	rs3117582	C	1.24 (1.16–1.33)	5.00 × 10^−10^	Wang, Y.^37^
		*MHC*	NSLC	rs2395185	T	1.17 (1.11–1.23)	9.50 × 10^−9^	Lan, Q.^21^
		*MHC*	SqCC	rs116822326	G	1.25 (1.19–1.32)	3.80 × 10^−19^	McKay, J.D.^40^
		*BAT2*	NSCLC	rs9469031	C	1.82 (1.52–2.17)	1.30 × 10^−10^	Jin, G.^23^
		*FKBPL*	NSCLC	rs200847762	G	4.00 (2.70–5.88)	9.80 × 10^−12^	Jin, G.^23^
	9p21.3	*CDKN2B-AS1*	LC	rs72658409	C	1.30 (1.19–1.39)	1.40 × 10^−10^	Wang, Z.^22^
			LC	rs1333040	C	1.14 (1.09–1.20)	2.30 × 10^−8^	Timofeeva, M.N.^38^
		*CDKN2A*	AC	rs885518	G	1.17 (1.11–1.23)	1.00 × 10^−9^	McKay, J.D.^40^
	12q13.13	*ACVR1B*	LC	rs11610143	C	1.12 (1.09–1.18)	5.00 × 10^−9^	Wang, Z.^22^
		*ACVR1B*	LC	rs12809597	T	1.39 (1.14–1.70)	1.20 × 10^−3^	Spitz, M.R.^42^
	15q25	*CHRNA3-CHRNA5*	LC	rs8034191	G	1.29 (1.23–1.35)	5.00 × 10^−20^	Hung, R.J.^31^
			AC	rs1051730	T	1.31 (1.27, 1.36)	1.91 × 10^−51^	Landi, M.T.^34^
			LC	rs55781567	G	1.30 (1.27–1.33)	3.10 × 10^−103^	McKay, J.D.^40^
			LC	rs2036534	T	1.39 (1.23–1.57)	2.30 × 10^−7^	Wu, C.^43^
			LC	rs667282	T	1.52 (1.35–1.71)	2.00 × 10^−12^	Wu, C.^43^
			LC	rs12910984	A	1.44 (1.28–1.63)	2.70 × 10^−9^	Wu, C.^43^
			LC	rs6495309	C	1.43 (1.27–1.61)	2.60 × 10^−9^	Wu, C.^43^
Chinese								
	1p36.32	*AJAP1-NPHP4*	NSCLC	rs9439519	G	1.11 (1.06–1.16)	3.65 × 10^−6^	Dong, J.^15^
	5q31.1	*IL3-CSF2-P4HA2-SLC22A5-ACSL6*	NSCLC	rs247008	G	1.12 (1.08–1.16)	7.68 × 10^−8^	Dong, J.^15^
	5q32	*PPP2R2B-STK32A-DPYSL3*	NSCLC	rs2895680	C	1.14 (1.09–1.19)	6.60 × 10^−9^	Dong, J.^15^
	6p21.1	*LRFN2*	LC	rs2494938	A	1.15 (1.08–1.22)	1.95 × 10^−6^	Jin, G.^20^
		*DQ141194*	LC	rs7741164	A	1.17 (1.12–1.22)	5.80 × 10^−13^	Wang, Z.^22^
	6q22.2	*ROS1-DCBLD1*	LC	rs9387478	G	1.18 (1.11–1.23)	4.14 × 10^−10^	Lan, Q.^21^
	6p21.32	*HLA class II*	LC	rs2395185	T	1.17 (1.11–1.23)	9.51 × 10^−9^	Lan, Q.^21^
	7p15.3	*DNAH11-SP4*	LC	rs2285947	A	1.17 (1.11–1.24)	1.57 × 10^−8^	Jin, G.^20^
	10p14	*GATA3*	NSCLC	rs1663689	A	1.14 (1.10–1.19)	2.84 × 10^−10^	Dong, J.^15^
	10q25.2	*VTI1A*	LC	rs7086803	A	1.28 (1.21–1.35)	3.54 × 10^−18^	Lan, Q.^21^
	12q23.1	*SLC17A8-NR1H4-SCYL2-GAS2L3*	SqCC	rs12296850	A	1.28 (1.19–1.39)	1.19 × 10^−10^	Dong, J.^19^
	13q12.12	*MIPEP-TNFRSF19*	LC	rs753955	G	1.18 (1.13–1.24)	1.50 × 10^−12^	Hu, Z.^14^
	17q24.3	*BPTF*	LC	rs7216064	A	1.16 (1.09–1.25)	6.59 × 10^−6^	Lan, Q.^21^
	20q13.2	*CYP24A1*	NSCLC	rs4809957	T	1.13 (1.08–1.18)	1.20 × 10^−8^	Dong, J.^15^
			NSCLC	rs2296239	C	1.18	5.95 × 10^−5^	Dong, J.^15^
	22q12.2	*MTMR3-HORMAD2*	LC	rs36600	A	1.29 (1.20–1.38)	6.20 × 10^−13^	Hu, Z.^14^
			LC	rs17728461	G	1.20 (1.14–1.27)	1.10 × 10^−11^	Hu, Z.^14^
Caucasian								
	1p31.1	*AK5*	LC	rs71658797	A	1.13 (1.09–1.18)	3.30 × 10^−11^	McKay, J.D.^40^
	2q32.1	*NUP35*	AC	rs11683501	G	1.17	1.60 × 10^−7^	Timofeeva, M.N.^38^
	6q27	*RNASET2-MIR3939*	LC	rs6920364	C	1.07 (1.05–1.10)	1.30 × 10^−8^	McKay, J.D.^40^
			LC	rs444210	A	1.07 (1.04–1.09)	3.61 × 10^−8^	McKay, J.D.^40^
	8p21.1	*CHRNA2*	LC	rs11780471	G	1.15 (1.10–1.20)	1.70 × 10^−8^	McKay, J.D.^40^
	8p12	*NRG1*	AC	rs4236709	G	1.13 (1.09–1.18)	1.30 × 10^−10^	McKay, J.D.^40^
	10q24.3	*OBFC1*	AC	rs11591710	C	1.16 (1.11–1.22)	6.30 × 10^−11^	McKay, J.D.^40^
	11q23.3	*MPZL2*	AC	rs1056562	T	1.11 (1.07–1.14)	2.76 × 10^−10^	McKay, J.D.^40^
	12p13.33	*RAD52*	SqCC	rs7953330	C	1.16 (1.11–1.20)	7.26 × 10^−13^	McKay, J.D^40^
				rs10849605	C	1.09	5.0 × 10^−7^	Timofeeva, M.N.^38^
	13q13.1	*BRCA2*	SqCC	rs11571833	T	1.84 (1.60–2.10)	1.84 × 10^−18^	Wang, Y.^39^
	15q21.1	*SECISBP2L*	AC	rs77468143	T	1.16 (1.12–1.20)	1.70 × 10^−16^	McKay, J.D.^40^
	20q13.33	*RTEL1*	AC	rs41309931	T	1.17 (1.11–1.23)	1.30 × 10^−9^	McKay, J.D.^40^
	22q12.1	*CHEK2*	SqCC	rs17879961	A	2.43 (1.92–3.13)	5.70 × 10^−13^	Wang, Y.^39^

^a^*LC* lung cancer, *NSCLC* non-small-cell lung cancer, *AC* adenocarcinoma of lung, *SqCC* squamous cell carcinoma of lung, *NSLC* lung cancer of never smoker

### Differences in susceptibility to adenocarcinoma and squamous cell carcinoma

Although most lung cancers occur due to smoking, the corresponding attributable fraction varies greatly by histological type; some types of lung cancer, such as SqCC, are thought to be almost exclusively due to smoking, whereas other types, such as AC, are thought to be less dependent on smoking. With decreased smoking prevalence and changes in tobacco composition, the distribution of histological types of lung cancer has undergone tremendous changes since the 1950s, and AC has overtaken SqCC as the most common type (40–50% *vs*. 20–30%) in Western countries.^44^ In China, over 70% of men were regular smokers at one point in their lives, whereas only 3% of women report having smoked regularly.^45^ The tobacco-attributed risk for the Chinese smokers was much less extreme than the risk for their counterparts in the West; for example, only a 2–4-fold increased risk of lung cancer was observed in the Chinese population versus the 20–30-fold increased risk observed among smokers in the West.^46^ The proportion of AC also increased from 25.93% (1995–1997) to 56.36% (2013–2015), exceeding the proportion of SqCC (the proportion of which decreased from 49.1 to 26.34%) and becoming the main histological type in the Chinese.

Notwithstanding the existence of several shared susceptibility loci between AC and SqCC (such as 8p21.1, 15q25, and 19q13) in the Caucasian population, there were significant differences in genetic susceptibility to AC and SqCC. Genetic loci associated with telomere length (such as 5p15, 10q24, and 20q13.33), somatic alterations (such as 8p12 and 9p21.3), or inflammation (15q21) were more evident in AC. In contrast, key players in the DNA damage response (such as 12p13.33-*RAD52*, 13q13.1-*BRCA2*, and 22q12.1-*CHEK2*) were more likely to be associated with susceptibility to SqCC, presumably because the products of these genes are needed to repair ongoing DNA damage that occurs on exposure to tobacco smoke.^40^ In the Chinese population, most of the identified susceptibility loci showed no histopathological differences, probably due to the relatively small sample size. However, evidence for negative interactions with smoking dose (such as 5q32) were more significant in AC, while positive interactions with smoking dose (such as 1p36.32) were more evident in SqCC.^13^

### Risk prediction based on the identified variants in a Chinese population

The application value of the identified variants for risk assessment of lung cancer was evaluated in recent study. GWAS dataset for lung cancer in a Chinese Han population were divided into a training set (Nanjing and Shanghai: 1473 cases, 1962 controls) and a testing set (Beijing and Wuhan: 858 cases, 1115 controls). A total of 14 variants with independent effects were used to build a genetic risk score (GRS). In the training set, the area under the curve (AUC) increased from 0.65 (0.63–0.66) to 0.69 (0.67–0.71) when the GRS were included in the risk prediction model; in the testing set, a similar improvement from 0.61 to 0.65 was observed.^47^ This result indicated that integrating traditional epidemiological risk factors through GRS was helpful for building the risk prediction model, a finding that may prove to be of great value in the identification of populations at high risk of lung cancer.

### The overall burden of somatic alterations in the genomes of Chinese lung cancer patients

Another hotspot of genomic studies on lung cancer has focused on somatic alternations (e.g., mutations, CNVs, and chromosome rearrangements). With the emergence of massively parallel sequencing technology, the genomic landscapes of cancer in European and American populations have been gradually deciphered by large cooperative projects such as The Cancer Genome Atlas^48^ (TCGA, launched in 2005) and The International Cancer Genome Consortium^49^ (ICGC, launched in 2009). In these studies, lung cancer is one of the few cancers with a high mutational burden because lung cancer patients commonly have a history of exposure to cigarette carcinogens.^50^ Recent whole-exome sequencing of specimens of malignant tissue from American lung cancer patients has revealed a median somatic mutation rate of 8.7 mutations/Mb and 9.7 mutations/Mb for AC and SqCC, respectively.^51^ These mutations were characterized by high proportions of cytosine-adenine (C to A) nucleotide transversions (the substitution of a purine for a pyrimidine or vice versa), which are seen predominantly in lung AC of smokers or ex-smokers rather than in lung AC of nonsmokers.^51–53^

In East Asians, the mutation rate of lung SqCC was similar to the rate observed in Americans (a mean mutation rate of 8.71 mutations/Mb).^54^ However, two Chinese studies on lung AC reported distinct mutation burdens. Wu, et al. reported a mutation rate of 9.7 mutations/Mb in 101 primary lung AC patients,^55^ while Li et al. observed a much lower mutation rate (1.4 mutations/Mb) in 271 lung AC patients.^56^ A study of whole-genome sequencing of NSCLC was conducted recently in the Nanjing Lung Cancer Cohort, providing a more accurate estimation of the mutation rate for the whole genome landscape.^57^ In this study, AC patients had a much lower mutation rate than SqCC patients (2.2 mutations/Mb for AC, 13.6 mutations/Mb for SqCC).^57^ Aside from possible technical reasons for the differences (differences in study design, sequencing methods, calling protocols, etc.), it is worth noting the gender difference in the patients of the two studies: Wu et al. included 39 female patients (38.6%),^55^ whereas Li et al. included 153 (56.5%).^56^ The lung cancer rates in Chinese women are higher than those in women in some European countries despite an extremely low prevalence of smoking.^58^ Second-hand smoke seems unlikely to be responsible for the female AC patients in the Chinese population from the point of view of genomics because these patients carry a low burden of smoking-induced mutations.^59^ Thus, the etiology of lung cancer in female patients without smoking behavior or elevated mutation rates remains largely unknown and warrants further investigation in future studies.

In addition to mutations, the large number of focal and broad areas that display somatic copy number alterations and genomic rearrangements illustrates the instability and complexity of the lung cancer genome.^51–55,60^ Intratumor heterogeneity can be inferred from chromosome instability and has been proven to be a prognostic predictor of lung cancer.^60^ Unfortunately, no similar study has been conducted in Chinese patients.

### Potential driver genes for lung cancer in Chinese patients

Although a great number of alterations occur in lung cancer genomes, the occurrence of only a few somatic alterations could serve as a direct trigger of cancer, imbuing cells with other oncogenic properties such as a growth advantage or immortal replication or activating invasion and metastasis and creating tumor-specific immune microenvironments, etc.^61^ These alterations commonly undergo positive selection in the evolution of cancer and can accumulate in the essential genes.^61^ Thus, genes that show mutation rates (or amplification/deletion rates) that are significantly higher than background are regarded as potential driver genes.^50^

**Fig. 1:**
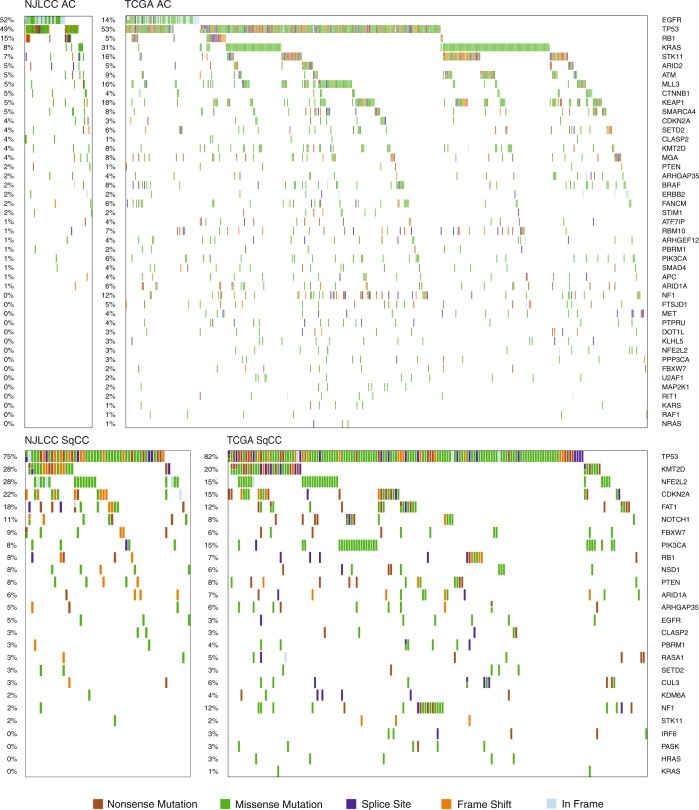
Mutation patterns of potential driver genes in the NJLCC and TCGA datasets. The data used to create this figure were taken from the study of Wang, C. et al.^57^ and the TCGA project^51,52^

In cases of lung AC, alterations usually affect oncogenes in the receptor tyrosine kinase/RAS/RAF pathway.^51,52,55,57^ Functional mutations in *EGFR* and *KRAS* are most common and are mutually exclusive. Interestingly, the mutation rate of *EGFR* has been found to be much higher in Chinese patients than in Americans (Chinese: 39–59% *vs*. TCGA: 14%), with a dominance of the L858R mutation, and *KRAS* is mutated more frequently in Americans (Chinese: 7–11% *vs*. TCGA: 31%). Notably, *EGFR* mutates more frequently in females and in nonsmokers.^55–57^ Other potential driver alterations in the pathway were shown to involve mutations (*BRAF*, *MAP2K1*, *HRAS*, *NRAS*, and *ERBB2*), exon skipping (*MET*) and gene fusions (*ROS1*, *RET*, and *ALK*), and their frequency was relatively low (<10%)^55–57^ in both populations. Among frequently mutated tumor suppressor genes identified in the TCGA data, the mutation rates of *TP53* (Chinese: 44% *vs*. TCGA: 53%), *STK11* (Chinese: 4–7% *vs*. TCGA: 16%), *KEAP1* (Chinese: 3–5% *vs*. TCGA: 18%) and *NF1* (Chinese: <2% *vs*. TCGA: 12%) were lower in Chinese patients (Fig. 1).^55–57^ In addition to the well-known amplified regions (3q26: *MECOM*; 5p15: *TERT*; 7p11: *EGFR*; 8q24: *MYC*; 11q13: *CCND1*; 12q13: *CDK4*; 12q15: *MDM2*; 14q13: *NKX2–1*) and deleted regions (17p13: *TP53*; 9p21: *CDKN2A/B*; 9p23: *PTPRD*), frequently amplified regions encompassing genes involved in cytoskeletal organization or focal adhesion, including *IQGAP3*, *TRIO*, *FSCN1*/*FSCN2*, *RAC1*/*RAC3*, and *ITGB4*/*ITGB8*,^55^ were identified in Chinese patients. Furthermore, highly activated *IQGAP3* was significantly associated with poorer overall survival of lung AC patients in a Chinese population.^55^

In contrast to lung AC, many tumor suppressors have been shown to be involved in SqCC.^51,53,54^ Kim et al. reported the first landscape of Asian SqCC in the Korean population. They found similarly high mutation rates in *TP53* (Korean: 73% *vs*. TCGA: 82%), *KMT2D* (Korean: 24% *vs*. TCGA: 20%), *NFE2L2* (Korean: 17% *vs*. TCGA: 15%), *NF1* (Korean: 12% *vs*. TCGA: 12%), and *PTEN* (Korean: 11% *vs*. TCGA: 8%) in Korean and TCGA patients. They also reported that *CDKN2A* mutations were infrequent in the Korean cohort (Korean: 4% *vs*. TCGA: 15%); however, mutations in *RB1* were more common (Korean: 7% *vs*. TCGA: 4%). Several shared amplified regions (3q26: *SOX2*; 8p11: *FGFR1*, *WHSC1L1*; 7p11: *EGFR*; 11q13: *CCND1*; 4q12: *KDR*, *KIT*, *PDGFRA*) and deleted regions (9p21: *CDKN2A*; 10q23: *PTEN*) were also identified. Unlike Korean patients, Chinese patients showed higher mutation rates in *NFE2L2* (28%) and *CDKN2A* (22%) and much lower mutation rates in *PIK3CA* (8%) and *NF1* (2%) (Fig. 1).^51,52,57^ These results suggest that Chinese patients may have genomic patterns of driver genes that are distinct from those of patients from other Asian countries.

### The great genomic heterogeneity between AD and SCC cells and genomics-driven precise lung cancer therapy for Chinese patients in the future

Considering the genome landscape of cancer cells mentioned above, the obvious heterogeneity between the AD and SCC genomes needed to be emphasized. AC could be described as a “gain-of-function” cancer, as most AC patients carry “gain-of-function” mutations or structural alterations in oncogenes involving the receptor tyrosine kinase pathway, such as L858R mutations in *EGFR* and *ALK*-*EML4* fusion genes. In most cases, these alterations have occurred recurrently, and the highly activated genes are ideal targets for molecular therapy. SCC patients, in contrast, are commonly affected by a great number of “loss-of-function” mutations in well-known tumor suppressor genes, such as frameshift or nonsense mutations in *TP53*, *PTEN*, and *CKDN2A*. The deficiency of tumor suppressor genes can also be attributed to the higher mutation rate in SCC. Smoking behavior may be one of the reasons for tumor suppressor loss in SCC patients. The heterogeneity of cancer genomics provides new insights into methods of treatment.

In the past decade, substantial progress has been made in the precise treatment of lung cancer, and this progress has benefited from the deep investigation of cancer genomics.^62^ Specific targeted agents against mutated EGFR and rearranged ALK have been successfully applied and have gradually become the standard first-line therapy for lung cancer.^63^ Due to the high mutation rate of *EGFR*, Chinese lung AC patients could benefit most from treatment with EGFR tyrosine kinase inhibitors (EGFR-TKI). However, the position of the mutation can dramatically influence the treatment outcome.^64^ For example, the benefit for tumors with exon 19 deletions was 50% greater than that for tumors with the L858R mutation, although L858R is the most prevalent mutation in Chinese patients.^64^ One possible reason for this could be the acquisition of resistance to first/second generation EGFR-TKI (e.g., Gefitinib, Afatinib) by tumor cells that carry the gain-of-function T790M mutation.^65^ Very recently, the third-generation EGFR-TKI Osimertinib, which targets T790M, was found to be effective against resistant NSCLC,^65^ bringing a new perspective to TKI treatment of lung cancer. However, most of these studies were conducted in Western populations; thus, a careful evaluation in Chinese patients is warranted. Another reason for EGFR-TKI resistance may be intratumor heterogeneity, since distinct subclones of tumor cells may carry different mutations. Thus, a comprehensive landscape of tumor heterogeneity in Chinese patients is necessary for further evaluation. More importantly, the genomic catalog of lung cancer is far from complete. The complexity of lung cancer poses a great challenge to investigators who are attempting to discover alterations that occur at low frequency (<10%) and to compare these alterations in Chinese and Western populations. The identification of cytoskeleton remodeling gene amplification^55^ is a good start and provides potential Chinese-specific targets for future therapy.

Another important process that has contributed to advances in treatment is the use of monoclonal antibodies (mAbs) and adoptive cellular therapy to treat cancer by modulating the immune response.^66^ Blockade of programmed cell death protein 1 (PD-1) has now been approved by the FDA for treatment of patients with NSCLC.^66^ One emerging biomarker of response to anti-PD-1 therapy is the tumor mutational burden.^67^ Thus, an effect of anti-PD-1 therapy in Chinese smoker patients is expected. Chinese female and never-smoker patients have a specific pattern of inflammatory lymphocyte infiltration that is mainly characterized by elevated numbers of B cells,^57^ indicating that advanced therapy that affects the inflammatory microenvironment in these patients should be emphasized and that it warrants future investigation.

### Interaction between germline and somatic changes in lung cancer

Emerging evidence indicates that the somatic evolution of a tumor may be significantly affected by inherited polymorphisms that are carried in the germline. By analyzing genomic data for 5954 samples from TCGA, Hannah et al. found 412 genetic interactions between germline polymorphisms and major somatic events across 22 cancer types.^68^ In recent analysis, lung cancer driver genes are observed more likely to be located within cancer susceptibility regions. The susceptibility variant rs36600 was associated with somatic mutations within *ARID1A*, the susceptibility variants rs2395185 and rs3817963 were associated with somatic alterations in the cell cycle pathway, and rs3817963 was associated with somatic alterations in the MAPK signaling pathway.^69^ These findings highlight the important role of germline-somatic interactions in tumorigenesis in lung cancer and help uncover the potential molecular mechanisms that underlie the GWAS findings. However, the majority of the samples used in the analysis were European; the results for the Chinese population have yet to be determined due to the lack of appropriate data.

## Outlook

Important progress had been made during the past decade in understanding host susceptibility and genomic alterations associated with lung cancer in the Chinese population. However, the way to recognize lung cancer is just starting at a new point. Missing heritability is still widespread in both Chinese and Caucasians.^70^ This indicates that further effort is needed to identify additional susceptibility loci for lung cancer. Compared to studies conducted in Caucasians, the sample sizes of GWAS in Chinese populations are relatively small.^41^ Additional 10 thousand pairs of lung cancer cases and matched controls are being genotyped in Chinese population in an effort to further characterize the genetic susceptibility to lung cancer. In addition, with the decreased cost of sequencing, it will soon be feasible to sequence whole-genome variants for thousands of samples simultaneously. The results of genome-wide gene-environment interaction studies will also help identify the missing heritability. With the initiation of large cohort studies in China, additional findings are foreseen in the near future.

The next important step is identification of the causal genetic variants and genes in the GWAS-reported loci and determination of the exact mechanisms of action of these variants through functional annotation or laboratory functional experiments. The results of the ENCODE Project and Roadmap Epigenomics provided a variety of annotation information (such as histone modifications and DNase hypersensitive sites) that is valuable for marking functional genomic segments in risk loci. Expression quantitative trait loci in a variety of tissues, such as the genotype-tissue expression,^71^ have proven to be a valuable resource for the functional study of GWAS loci. The TCGA project provided much more dimensional data, including gene expression, genotype, epigenetics, and somatic alterations as well as the proteome, that constitute a precious resource for research on the biological mechanism of lung cancer.^52,53^ With these public resources, several candidate susceptibility genes, including *RNASET2* in 6q27, *NRG1* in 8p12, *AMICA1* in 11q23.3, and *SECISBP2L* in 15q21.1, have been identified through functional annotation. However, most of the samples used in these resources were obtained from Caucasians, and the mechanisms of action of the specific susceptibility loci of lung cancer in the Chinese population are still unclear. With the accumulation of multiomics data on lung cancer in the Chinese population, more meaningful findings are foreseen in the near future.

Therefore, findings from genetic associations and somatic alterations are not only important in elucidating the etiology of lung cancer but are also critical to the clinical targeted therapy of lung cancer. This genetic knowledge will provide an important foundation for the development and improvement of future clinical strategies for lung cancer precision oncology.

## Data Availability

The data used to create Fig. 1 were taken from the study of Wang C et al.^57^ and the TCGA project.^51,52^
